# Innovating Pelvic Fracture Surgery: Development and Evaluation of a New Surgical Table for Enhanced C-Arm Imaging and Operational Efficiency

**DOI:** 10.3390/jcm14093169

**Published:** 2025-05-03

**Authors:** Yong-Cheol Yoon, Min Jun Kim, Ji Sub Lim, Hyung Keun Song

**Affiliations:** 1 Orthopedic Trauma Division, Trauma Center, Gachon University College of Medicine, Namdong-gu, Incheon 21565, Republic of Korea; dryoonyc@gmail.com (Y.-C.Y.); himman03@naver.com (M.J.K.); 2 Department of Orthopedic Surgery, Gachon University College of Medicine, Namdong-gu, Incheon 21565, Republic of Korea; 18935@gilhospital.com; 3 Department of Orthopaedic Surgery, Ajou University School of Medicine, Yeongtong-gu, Suwon-si 16499, Gyeonggi-do, Republic of Korea

**Keywords:** C-arm imaging, cost-effectiveness, operating table, pelvic fracture surgery, time-saving

## Abstract

**Background:** Pelvic fractures require precise reduction and stabilization, necessitating high-quality C-arm imaging and accurate patient positioning. Standard operating tables often obstruct optimal C-arm maneuverability. To address this, we developed a new auxiliary surgical table that integrates with existing tables and evaluated its clinical utility compared to a specialized carbon surgical table. **Methods:** Between March 2018 and June 2023, we conducted a retrospective study involving 162 patients (97 men and 65 women; average age 45.7 years) who underwent percutaneous sacroiliac screw fixation for pelvic fractures. Ninety patients were treated using the newly developed table, and seventy-two patients were treated using the carbon table. The new table, measuring 200 cm in length, 50 cm in width, and 2 cm in thickness, was constructed from waterproof plywood and designed to be securely attached to existing operating tables. We compared surgical preparation times, economic costs, and intraoperative imaging feasibility between the two groups. **Results:** Use of the new table significantly reduced the surgical preparation time by an average of 21 min and saved approximately $43,000 in cost compared to the carbon table. Subjective assessments indicated no notable difference in intraoperative C-arm image quality between the two groups. The new table allowed free C-arm rotation by overcoming the mechanical limitations of conventional tables. **Conclusions:** The new auxiliary table demonstrated clinical feasibility and economic advantages without compromising intraoperative imaging quality, offering a practical and cost-effective alternative for pelvic fracture surgeries.

## 1. Introduction

Unlike long bones, the pelvic bone possesses a three-dimensional structure and contains various organs, crucial blood vessels, and nerves [1]. For proper reduction and stabilization, high-quality C-arm imaging, the correct patient position, and the choice of surgical table are essential [2]. Pelvic ring injuries often require minimally invasive percutaneous fixation techniques, particularly in elderly patients with comorbidities, where open reduction can increase the risk of complications [3]. Considering the patient’s condition and fracture type, minimally invasive percutaneous screw fixation is increasingly used, especially in older patients with chronic diseases and a high risk of complications, necessitating sophisticated surgeries.

**Figure 1:**
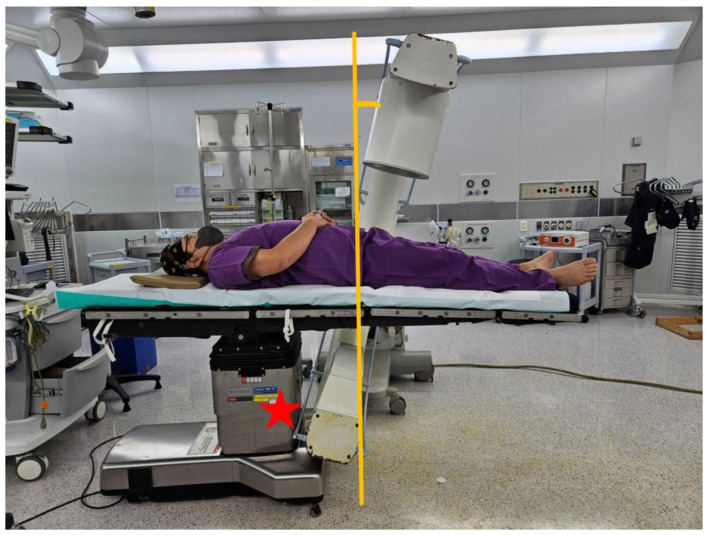
The conventional operating table, with its columns centrally located, restricts the movement of the C-arm during pelvic bone fracture surgeries, preventing the capture of a clear pelvic outlet view (red star).

A specialized surgical table for pelvic fractures, allowing free C-arm movement in multiple directions, is required [4]. However, standard surgical tables, while facilitating surgeries for upper- and lower-limb fractures, present a significant challenge for pelvic surgeries. Especially, the central column’s placement coincides with the patient’s pelvic area, obstructing proper C-arm imaging. Additionally, capturing a pelvic outlet view requires tilting the C-arm’s X-ray tube toward the patient’s head. The surgical table’s column obstructs rotation, hindering the possibility of obtaining a clear view of the pelvic outlet. Consequently, conventional surgical tables are highly unsuitable for pelvic surgeries (Figure 1).

**Figure 2:**
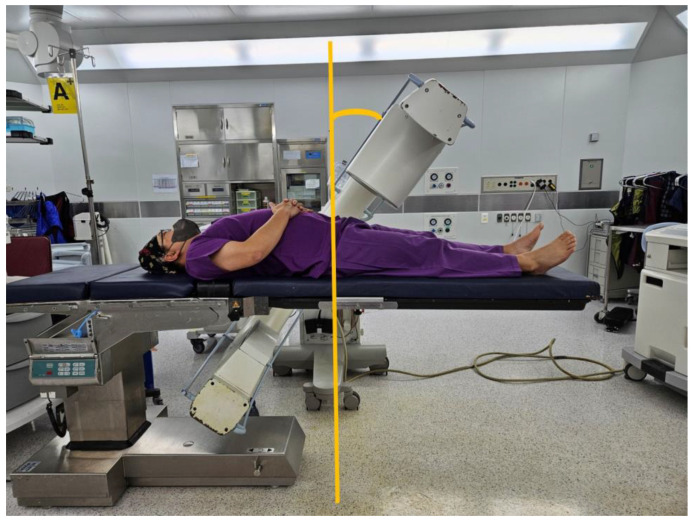
The carbon table, with its columns eccentrically arranged, allows for an unobstructed view of the pelvic outlet during pelvic bone fracture surgeries. Although the design of the table is beneficial for such operations, it is important to note that the table comes with a high purchase cost, and its installation requires a significant amount of time.

To address this issue, a table with an eccentric column near the patient’s head has been developed. This particular table features a carbon-made contact area for the patient, allowing unobstructed C-arm movement and improved radiolucency (Figure 2). However, this table’s cost exceeds $100,000, making it less affordable. In addition, the relatively low incidence of pelvic fractures compared to other orthopedic conditions often limits the cost-effectiveness of such specialized equipment in general trauma centers [5].

To address the clinical need for a more cost-effective and time-efficient approach in pelvic fracture surgery, we developed an auxiliary table that can be attached to existing surgical tables. This design aims to overcome specific limitations of conventional tables, particularly those that restrict C-arm access and complicate intraoperative imaging. In this study, we evaluated the new table with respect to intraoperative image quality, surgical preparation time, cost-effectiveness, and clinical feasibility.

## 2. Materials and Methods

### 2.1. Patients

Between March 2018 and June 2023, a retrospective study was conducted at a single level 1 trauma center involving 162 patients diagnosed with pelvic fractures. The study population consisted of 97 men and 65 women, with an average age of 45.7 years. All patients underwent percutaneous sacroiliac screw fixation as the primary surgical intervention. The patients were divided into two groups based on the type of surgical table used: 90 patients were operated using a newly developed auxiliary table (new table group), while 72 patients were operated using a specially designed carbon fiber surgical table (carbon table group). Patient allocation was non-randomized and primarily determined by the availability of the tables during the study period. The study design and data collection were approved by the Institutional Review Board of the Human Experimental and Ethics Committee of our hospital (IRB No. GBIRB2024-013). The patients and/or their families were informed that data from their cases would be submitted for publication and provided their consent.

All procedures were performed at a single level 1 trauma center by three orthopedic surgeons. Among them, two surgeons had over 10 years of clinical experience in pelvic and acetabular trauma, while one was a junior surgeon with under 3 years of experience. This composition ensured that the evaluation incorporated diverse levels of surgical expertise.

### 2.2. Surgical Methods

**Figure 3:**
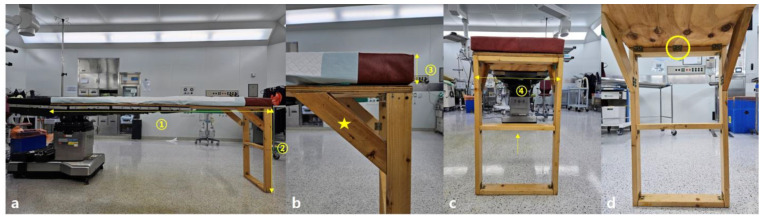
(**a**) Frontal clinical photograph of the new integration method with the existing surgical setup. The new table measures a length (①) of 220 cm and a height (②) of 83 cm. (**b**) A close-up of the corner detailing the added triangular (star-shaped) supports enhancing structural rigidity, alongside a 7 cm thick mattress (③) matching the firmness of conventional options. (**c**) The side profile reveals a width (④) of 50 cm, consistent with existing table, reinforced by a central wooden brace (arrow) to reduce instability. (**d**) The interior perspective shows all joins neatly fastened with hinges (circle).

The newly manufactured table has a support base width of 50 cm and a length of 220 cm, with a 2 cm thickness. The table is used in conjunction with the support base of an existing surgical table (Figure 3).

The material of the new table’s support base is fireproof and waterproof plywood, consisting of seven layers with the grain of each layer arranged perpendicularly to each other [6,7]. The table has two legs made of ash wood, each 81 cm long and 10 × 3 cm in thickness, connected using hinges and further stabilized by a central triangular support. The wood is easily available in the market and is commonly used for making furniture, such as desks, wardrobes, and beds [8].

**Figure 4:**
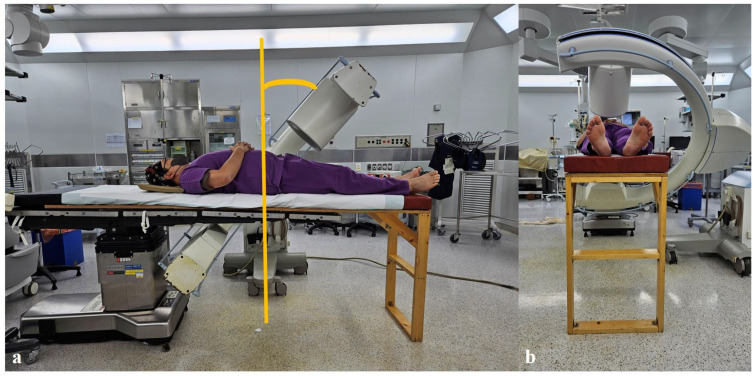
An innovative approach involves combining a newly developed table made of plywood with the existing surgical table. (**a**,**b**) This configuration allows the patient to securely place their legs below the knee joint on the new table, thereby facilitating unrestricted viewing of the pelvic outlet view during surgery.

The height of the existing table is first adjusted to align with the new table. Then, the new table is combined with the existing one, ensuring that more than two-thirds of the new table’s support base overlaps with the existing table for stability. At least four-fifths of the patient’s weight is positioned on the existing table, including the head, chest, abdomen, and distal femur area, while the new table provides support from below the knee joint to enhance stability (Figure 4).

Ensuring no gap exists where the new and existing tables meet is crucial, as any separation could result in movement and overall instability. As the new table is not height-adjustable, surgeons are advised to use a footrest for height adjustment. Depending on the desired view, the surgeon can stand on the footrest or step down for precision. A mattress of suitable height is prepared to help align the new table with the existing one. The mattress should not be too hard to prevent numbness due to anesthesia or too soft to avoid the lower limbs sinking into it. The used mattress has similar firmness to the one used on the existing table, with a height of 7 cm.

To minimize concerns regarding hygiene and sterilization, a mattress was placed over the plywood surface, and the fabric covering the mattress was regularly disinfected. Additionally, two layers of sterile surgical drapes were applied during each operation in an effort to completely isolate the patient from direct contact with the plywood platform.

### 2.3. Evaluation

This study evaluated and compared the practicality, cost-effectiveness, stability, and time management aspects between the new table group and the carbon table group. First, practicality was assessed by comparing the C-arm imaging capabilities of the two groups. The pelvic outlet view angles were measured with the patient in the supine position on each table, and the angles required to achieve clear visualization of the S1 and S2 sacral foramina were recorded [9]. These measurements were obtained under fluoroscopic guidance using a digital inclinometer integrated into the C-arm system. Intraoperative image quality was subjectively assessed by the operating surgeons, based on the clarity and visibility of the sacral foramina and surrounding pelvic landmarks.

Additionally, surgical preparation time was measured by recording the interval from the start of the operating-table setup—including installation, patient positioning, and C-arm alignment—to the moment of skin incision. This information was collected retrospectively from the operative and nursing records for both groups.

Economic aspects were evaluated by calculating and analyzing the manufacturing cost of the new table compared to the purchase cost of the carbon table. For stability assessment, the biomechanical capacity of the new table was tested by sequentially placing dumbbells (10 kg each, up to a total of 50 kg) on the table surface and visually observing for any bending or twisting.

### 2.4. Statistical Analysis

Statistical analysis in this study was conducted using SPSS 25.0 (IBM Corp., Armonk, NY, USA). Key variables, such as angle measurements and surgery preparation times, were classified as continuous variables. The normality of these variables was verified through a Kolmogorov–Smirnov test. Variables displaying a normal distribution were expressed in terms of mean and standard deviation, while those not following a normal distribution were described using median and range. For analyzing mean differences between the two groups, an independent samples T-test was used for normally distributed continuous variables, and a Mann–Whitney U Test was used for non-normally distributed variables. The level of significance was set at *p*-value < 0.05. In addition to statistical significance, the results were interpreted in light of clinical utility and feasibility, especially given the study’s retrospective and single-center nature.

## 3. Results

The baseline demographic and clinical characteristics were comparable between the new table group and the carbon table group, without any statistically significant differences (Table 1).

In a total of 162 pelvic fracture surgeries, the average preparation time using the new table methods was 12 ± 8 min, compared to 33 ± 15 min when preparing with the carbon table, resulting in a significant reduction of 21 min in surgery preparation time (*p* < 0.001, Table 2).

The pelvic outlet view angles were comparable between the two groups. The average pelvic outlet view angle was 58.7° (range, 51–63°) in the new table group and 57.0° (range, 50–62°) in the carbon table group, with no statistically significant difference (*p* = 0.42). Both tables allowed clear visualization of the sacral foramina under fluoroscopic guidance, facilitating accurate screw placement.

In terms of cost, the new table was manufactured for $700, whereas the specially designed carbon table for pelvic fractures costs $50,000. This resulted in a substantial saving of $43,000. From a safety perspective, no safety incidents have been reported since 2018, indicating the new table’s stable use in surgeries. Additionally, in load-bearing tests where 10 kg dumbbells were placed on the wooden table, the table demonstrated no signs of warping even with five dumbbells, suggesting that it could easily support a patient weighing 150 kg, assuming that both lower legs constitute 10–15% of a person’s total body weight [10]. Radiolucency was subjectively assessed by visual comparison of intraoperative C-arm images, and no significant differences were noted between the new table and the carbon table throughout the study.

During the study period, four cases of deep pelvic infection were observed, with similar incidence in both groups. Due to the non-randomized design, no causality could be established. They were successfully treated with massive debridement and irrigation without the need for plate removal.

## 4. Discussion

This study revealed that pelvic fracture surgeries, which posed significant challenges when conducted on traditional standard tables with a central column, became much more manageable when performed with the new table. The new table allows for free movement of the C-arm and easier exposure of the pelvic area, facilitating an effective surgery. In particular, the pelvic outlet view could be more easily obtained, and the sacral foramen was clearly visible during fluoroscopy, which is critical for accurate screw placement. Additionally, the preparation time was significantly reduced by approximately 21 min, as confirmed by intraoperative timing records, since the new table is attached to the existing one rather than replacing the entire surgical setup. This time saving translates to improved operating room turnover and procedural efficiency [11].

Economically, this method is also advantageous, as it costs only $700—substantially less than a carbon table costing around $50,000—offering a highly cost-effective alternative [12]. This may be especially useful in facilities with limited resources or those performing pelvic surgeries infrequently. However, emphasizing that the new table is an adjunct to the surgical table and should not bear more than half of the patient’s weight to maintain stability is essential. Bench testing demonstrated structural stability under controlled load, and clinical use since 2018 has not resulted in any safety-related incidents.

With the increase in pelvic and acetabular fractures due to medical advancements, industrialization, and an aging population, a growing need for surgical interventions has been observed [4,13]. The complexity of the pelvis, with its three-dimensional structure, intertwined blood vessels and nerves, and internal organs, necessitates precise and meticulous surgery [1]. Surgical methods are evolving from open reduction and internal fixation to minimally invasive plate osteosynthesis (MIPO). MIPO is preferred in situations where both procedures are viable due to its comparable fixation strength, less physical trauma to patients, reduced blood loss, and short operation time [14]. Optimal surgical beds and C-arm equipment are essential to meet these surgical demands and requirements [5].

In the past, when pelvic fracture surgeries were less common, tables with a central column were advantageous for surgeries on limbs, as they helped maintain the center of gravity. However, as pelvic surgeries are performed around the center of the table, a specially designed bed with an eccentric column, often attached at the head and made of carbon, is used (Figure 2). Carbon tables, despite their high cost, are necessary, owing to their excellent radiolucency, reducing radiation exposure for both medical staff and patients [5]. Our newly developed auxiliary table also provided a radiolucent operative field, and intraoperative imaging revealed no noticeable obstruction or artifact compared to the carbon table [15]. Although formal radiodensity measurements were not performed, surgeons reported comparable image clarity for procedural guidance.

The method employed in this study involves connecting a device to an existing bed, with a production cost of approximately $700. This approach is economically advantageous and may offer similar practical benefits to a carbon table, although no direct comparative analysis was performed. Moreover, even if a hospital possesses a carbon table, the necessity to move the table for surgical preparation can be time-consuming. In contrast, our new table design, which attaches to the existing one, eliminates the need for relocation, thereby significantly reducing the preparation time for surgery. These benefits were demonstrated using formal data collection for setup time and intraoperative fluoroscopic positioning, ensuring that the results reflect measurable performance, not just subjective impressions.

The primary concern regarding the new table installation is its stability and durability. Concerns may arise regarding placing an anesthetized patient on a wooden table. However, as demonstrated, most of the patient’s weight, including the head, chest, abdomen, and pelvic area, is supported by the existing table, with the wooden table supporting the area below the knee (Figure 4). The wooden table, made of internally reinforced waterproof plywood and supported by a triangular structure with hinges, has been used effectively since 2018. Mechanical tests have also confirmed the ability of this table to support a load of up to 50 kg. Nonetheless, clinicians should remain cautious and ensure correct positioning to prevent mechanical overload.

Another concern with the new table is infection control. Sterile drapes were used to prevent direct contact between the patient’s body and the table, with clear demarcation of the surgical area [16]. The plywood’s vulnerability to water was addressed by adding waterproofing during its manufacture. Infections during the study period were attributed to patient conditions or the severity of injuries, not to the table.

As aseptic techniques and surgical procedures have advanced, surgical tables have also evolved from static to dynamic, accommodating various procedures [17]. With the advent of C-arm imaging for orthopedic surgeries, surgical tables now incorporate radiolucent materials for anatomical imaging of bones. However, with advancements come high costs, making these tables unaffordable for some hospitals or junior surgeons [18]. In such cases, the method described in this study can be a reliable, easy, safe, and cost-effective alternative for performing pelvic fracture surgeries and obtaining appropriate imaging.

This study has several limitations that should be acknowledged. The new table has not undergone formal biomechanical or sterilization testing, which limits its regulatory applicability. Although preventive measures were implemented—such as placing a disinfected 7 cm thick mattress over the plywood surface and applying two layers of sterile surgical drapes during each procedure—the inherent properties of plywood prevent achieving the high-level sterilization standards required in operating room environments. As an auxiliary structure, the new table must be combined with the primary operating table and is intended to support only the lower extremities, without independent height adjustment, occasionally requiring the use of a footrest by surgeons to optimize positioning. Additionally, the study was conducted at a single center with non-randomized group allocation, and although baseline characteristics were analyzed statistically, complete control of baseline comparability could not be guaranteed. No direct objective radiographic comparison between the new table and the carbon table was performed, and radiolucency was assessed subjectively by the operating surgeons rather than through standardized imaging analysis. These limitations reduce the generalizability and evidential strength of the findings. Future studies should aim to include prospective, randomized, multicenter designs with objective radiographic assessments, and further development of the auxiliary table should focus on using fully sterilizable, medical-grade materials to meet infection control standards.

## 5. Conclusions

The newly developed auxiliary surgical table, designed to be combined with existing operating tables, demonstrated significant improvements in surgical preparation time, C-arm accessibility, and cost-efficiency in pelvic fracture surgery. Despite the limitations of a single-center, retrospective design, the results suggest that this approach is both clinically practical and economically advantageous. Further prospective, multicenter studies are warranted to confirm these findings and expand their applicability in diverse clinical settings.

## Figures and Tables

**Table 1:** Demographic and clinical characteristics of patients.

Characteristics	Carbon Table (*n* = 72)	New Table (*n* = 90)	*p*-Value
Sex (Male/Female)	43/29	54/36	0.95
Age (years, mean ± SD)	46.0 ± 11.8	45.5 ± 12.1	0.74
BMI (kg/m^2^, mean ± SD)	24.7 ± 3.6	24.3 ± 3.2	0.68
Injury mechanism (Traffic accident/Fall/Other)	38/29/5	48/35/7	0.82
ASA classification (I/II/III)	23/42/7	30/50/10	0.92
ISS (mean ± SD)	8.5 ± 3.5	8.1 ± 3.2	0.63

**Table 2:** Comparative analysis of carbon and new table in pelvic fracture surgeries.

Indicator	Carbon Table (*n* = 72)	New Table (*n* = 90)	*p*-Value
Surgery preparation time (min)	33 ± 15	12 ± 8	<0.001
Pelvic outlet view angle (°)	57.0 (50–62)	58.7 (51–63)	0.42
Subjective image quality	Good	Good	
Table cost (USD)	$50,000	$700	

## Data Availability

The datasets generated and/or analyzed during the current study are not publicly available because of restricted access to our hospital database but are available from the corresponding author upon reasonable request.

## Abbreviation

The following abbreviation is used in this manuscript:

MIPO	Minimally invasive plate osteosynthesis
BMI	Body mass index
ASA	American Society of Anesthesiologists
ISS	Injury Severity Score
